# Emotion Regulation and Attachment Style as Predictors of Psychiatric Hospitalization Duration in Suicidal Adolescents

**DOI:** 10.3390/children13040448

**Published:** 2026-03-25

**Authors:** Einav Isack, Shiri Ben-David, Tanya Goltser-Dubner, Ronen Segman, Ella Kianski, Ruth Giesser, Shlomo Rahmani, Pnina Blum Weinberg, Amichai Ben-Ari, Yaron Sela, Moriah Bar Nitsan, Amit Lotan, Tanya Schechter, Moshe Daninos, Shai Yishai, Yael Avraham, Fortunato Benarroch, Amit Shalev

**Affiliations:** 1 Department of Psychology, Hebrew University of Jerusalem, Jerusalem 91905, Israel; einav.isack@mail.huji.ac.il (E.I.); shiri.ben-david@mail.huji.ac.il (S.B.-D.); 2 Department of Psychology, Hadassah Hebrew University Medical Center, Jerusalem 91120, Israel; 3 Molecular Psychiatry Laboratory, The Herman-Danna Division of Pediatric Psychiatry, Department of Psychiatry, Hadassah Medical Organization, Faculty of Medicine, Hebrew University of Jerusalem, Jerusalem 91240, Israel; tanyagol@hadassah.org.il (T.G.-D.); ronense@ekmd.huji.ac.il (R.S.); 4 The Herman-Danna Department of Pediatric Psychiatry, Hadassah Medical Organization, Faculty of Medicine, Hebrew University of Jerusalem, Kiryat Hadassah, Ein Kerem, Jerusalem 91120, Israel; elak@hadassah.org.il (E.K.); rutig@hadassah.org.il (R.G.); moriabn@hadassah.org.il (M.B.N.); mdaninos@hadassah.org.il (M.D.); shaiyi@hadassah.org.il (S.Y.); yaelahrak@hadassah.org.il (Y.A.); fortuben@hadassah.org.il (F.B.); 5 Adult Inpatient Unit, The Biological Psychiatry Laboratory, Hadassah Medical Organization, Faculty of Medicine, Hebrew University of Jerusalem, Jerusalem 91120, Israel; shlomo80@hadassah.org.il (S.R.); amitlo@hadassah.org.il (A.L.); 6 The Donald Cohen Child and Adolescent Psychiatry Department, Eitanim Psychiatric Hospital, The Jerusalem Mental Health Center, Jerusalem 90805, Israel; pnina.b@moh.gov.il; 7 Department of Behavioral Sciences, Ariel University, Ariel 40700, Israel; 8 The Research Center for Internet Psychology (CIP), Reichman University, Herzliya 46150, Israel; yaron.sela02@post.runi.ac.il; 9 Eitanim Psychiatric Hospital, The Jerusalem Mental Health Center, Jerusalem 90805, Israel; tanya.shechter@psjer.health.gov.il

**Keywords:** adolescent, suicidal ideation, emotion regulation, attachment style, inpatient care

## Abstract

**Highlights:**

**What are the main findings?**

- Greater difficulties in emotion regulation are associated with longer psychiatric hospitalization among suicidal adolescents, independent of depression, anxiety, and suicidality severity.
- Avoidant attachment is associated with shorter hospitalization and partially mediates the association between emotion regulation difficulties and length of stay.

**What are the implications of the main findings?**

- Emotion regulation and attachment style are important factors in understanding individual differences in psychiatric hospitalization duration among suicidal adolescents.
- Integrating emotion-focused and attachment-informed approaches into inpatient care may enhance treatment engagement and support more appropriate hospitalization duration.

**Abstract:**

Background: Emotion regulation and attachment styles are interrelated and are critical factors in psychopathology and treatment outcomes, particularly in youths with suicidal behavior receiving psychiatric inpatient care. This study examined the influence of emotion regulation and attachment style on psychiatric hospitalization duration among adolescents admitted due to suicidal ideation or behavior. Methods: Participants included 79 Israeli adolescents (mean age 15.35 years, 87.3% female) admitted to a tertiary psychiatric inpatient unit following a suicidal crisis. Data was collected using the Difficulties in Emotion Regulation Scale (DERS), the Experience in Close Relationships Scale (ECR), the Screen for Child Anxiety-Related Emotional Disorders (SCARED), the Patient Health Questionnaire (PHQ-9), and the Columbia-Suicide Severity Rating Scale (C-SSRS). Data were analyzed using correlation and multiple regression analyses. Results: Analysis revealed that greater emotion regulation difficulties predicted longer hospitalization duration (β = 0.41, *p* < 0.001), while avoidant attachment style was associated with shorter hospitalization duration (β = −0.35, *p* < 0.001). Notably, the level of suicidality as well as psychopathology symptoms (depression and anxiety) did not predict hospitalization duration. Conclusions: These findings underscore the important role of emotion regulation and attachment style in determining treatment duration in suicidal adolescents, beyond the severity of psychopathology and suicidality, suggesting their unique contribution to treatment planning. Clinical interventions targeting emotion regulation and attachment styles could enhance inpatient care effectiveness, offer a more personalized treatment approach and potentially reducing hospitalization duration.

## 1. Introduction

Emotion regulation has been extensively studied in various psychological contexts. Despite its prominence in the literature, a clear and universally accepted definition remains elusive [1,2,3]. Some researchers conceptualize emotion regulation as the processes through which individuals manage and express their emotional states [4]. Others emphasize the goal-directed aspect of regulation, suggesting that it involves efforts to influence emotional responses in accordance with internal or external objectives, highlighting the roles of emotional awareness, regulatory goals, and strategies [5].

There is general consensus regarding the classification of emotion regulation strategies into adaptive and maladaptive categories. Adaptive strategies, such as cognitive reappraisal, acceptance, and problem-solving, are typically associated with positive psychological outcomes. Reappraisal involves reframing an event to diminish its negative emotional impact; acceptance refers to acknowledging emotions without judgment; and problem-solving entails actively addressing distressing situations [1,6].

In contrast, maladaptive strategies include rumination, suppression, and avoidance. Rumination is the repetitive focus on negative emotions and their causes without moving toward resolution. Suppression involves efforts to push distressing thoughts and feelings out of awareness, and avoidance encompasses both cognitive and behavioral disengagement from distressing stimuli [1,6].

Emotion regulation strategies are significantly shaped by early interpersonal interactions, particularly those involving caregivers. Inconsistent, neglectful, or abusive caregiving environments can impede the development of effective regulatory skills, leaving children to adopt maladaptive strategies in response to emotional challenges [7,8]. Such maladaptive patterns heighten vulnerability to various psychopathologies, including depression, anxiety, and borderline personality disorder [6,9].

The bidirectional relationship between emotion regulation and psychopathology is well-established: while emotion regulation difficulties can contribute to the onset of psychological disorders, psychopathology itself can further impair regulatory capacities by altering emotional reactivity (e.g., hyper- or hypo-reactivity) and promoting rigid or maladaptive regulation strategies [10,11]. Effective emotion regulation is context-dependent and involves the flexible application of strategies appropriate to specific situations [12,13]. Notably, strategies such as avoidance and suppression are particularly associated with heightened anxiety, depression, and disordered eating, whereas problem-solving and cognitive reappraisal serve as protective factors [6,14]. Beyond reducing psychopathology, effective emotion regulation has also been associated with greater psychological well-being, resilience, and adaptive functioning across developmental stages [6,10,11].

Attachment theory, initially proposed by John Bowlby [15], emphasizes the importance of early caregiver–infant bonds in shaping emotional and behavioral development. These attachment patterns are internalized as working models that influence emotion regulation and interpersonal behavior throughout life [16]. Modern research conceptualizes attachment on two continuous dimensions: anxious and avoidant [17,18].

Anxiously attached individuals are preoccupied with fears of abandonment and display heightened vigilance toward relational threats, often employing hyperactivating strategies like rumination [19]. Conversely, avoidantly attached individuals prefer emotional distance, often utilizing deactivating strategies such as suppression and avoidance [20]. Both attachment patterns have been linked to emotion regulation difficulties and increased psychopathological risk [20,21,22]. These associations are thought to arise from internal working models formed through early caregiving experiences. When caregivers are inconsistently available or unresponsive, children may not reliably experience effective co-regulation, which is crucial for learning to modulate arousal and soothe distress. Over time, this can shape their appraisal of emotional cues, leading to heightened vigilance, difficulty tolerating negative affect, and reliance on rigid or maladaptive regulation strategies, such as rumination or suppression, in response to emotional challenges [20].

One of the major risks caused by psychopathology is suicidality, being one of the leading causes of death among adolescents worldwide, and ranked as the third leading cause of death among individuals aged 15–29 years [23]. In Israel, according to a national data report, in 2020, the age-adjusted suicide rate was 6.4 per 100,000 individuals, alongside 6861 emergency department presentations due to suicide attempts in 2022, with notable increases in attempt rates among youth aged 10–17 over the past decade [24]. Established risk factors for adolescent suicidal thoughts and behaviors include mood disorders such as depression and anxiety, previous suicide attempts, substance use and misuse, and psychosocial stressors, including bullying and family-related difficulties [25].

Emotion regulation difficulties have been identified as a critical factor in suicidality. Adolescents with limited access to effective regulatory strategies are more likely to report suicidal ideation and attempts [26,27]. Emotion regulation difficulties are often accompanied by heightened impulsivity and low distress tolerance, which not only increases psychological pain and suicidal intent but also may convert ideation into behavior [28,29]. Insecure attachment patterns further compound this risk. Several studies have demonstrated that adolescents with anxious or avoidant attachment are more prone to suicidal behaviors [30,31,32,33]. Insecure attachment may reflect a broader vulnerability stemming from impaired relational security and emotion dysregulation.

Emotion regulation difficulties and suicidality are closely linked. Regulatory vulnerability may become more pronounced under conditions of prolonged social restriction and diminished interpersonal contact. Empirical findings from the COVID-19 pandemic have shown that lockdown-related isolation was associated with elevated feelings of loneliness, greater emotional distress, and increased suicidal ideation among adolescents, illustrating how broader environmental stressors can interact with individual regulatory capacities to heighten suicide risk [34]. In addition, online victimization, which can also influence emotion regulation, has emerged as a relevant psychosocial risk factor for adolescent suicidality [35].

Psychiatric inpatient care provides adolescents with intensive, multidisciplinary treatment aimed at stabilizing acute crises, including suicidality. Evidence suggests that inpatient interventions are generally effective in promoting clinical improvements that can be sustained post-discharge [36,37,38]. However, hospitalization carries inherent risks. Adolescents may experience emotional distress from witnessing the struggles of their peers, potentially leading to symptom contagion or the adoption of maladaptive behaviors [39,40,41,42]. Moreover, inpatient care disrupts adolescents’ natural routine and may distance them from crucial social support systems, such as family and school [43]. These factors necessitate a careful balance between the therapeutic benefits of hospitalization and its potential unintended consequences.

The ability to regulate emotions effectively may facilitate more adaptive engagement with treatment and a smoother adjustment to the inpatient environment, potentially improving treatment outcomes by enabling patients to tolerate distress, maintain goal-directed behavior, and effectively apply therapeutic strategies [2,44,45,46]. Conversely, emotion regulation difficulties may hinder distress management and the ability to benefit from therapeutic interventions, potentially resulting in prolonged and more challenging inpatient treatment processes [47].

In acute adolescent psychiatric settings, discharge decisions are typically based on clinical stabilization, including reduction in suicidal risk and sufficient emotional and behavioral regulation to allow safe return to the community. Thus, although length of stay is influenced by systemic and contextual factors, in structured inpatient settings, it mainly reflects the degree of psychological stabilization required prior to discharge. Importantly, prolonged hospitalizations in youth have been associated with more severe trajectories of suicidal ideation and increased risk of rehospitalization and out-of-home placement [48,49], underscoring the clinical relevance of understanding psychological factors that contribute to hospitalization duration. Length of stay should therefore be conceptualized as a clinically determined outcome that may be influenced by multiple factors and reflects the course of inpatient treatment rather than a purely psychological indicator.

Given the central role of emotion regulation in the development and maintenance of suicidal behavior, and its established association with psychological functioning and symptom severity, it is plausible that regulatory difficulties may influence the course of inpatient treatment [10,12]. Similarly, attachment orientations, which shape patterns of help-seeking, interpersonal trust, and affect regulation, may affect how adolescents engage with therapeutic environments [20]. Therefore, examining the combined and interactive roles of emotion regulation difficulties and attachment patterns in predicting hospitalization duration may offer clinically meaningful insights. The aim of the present study is to examine the association between emotion regulation, attachment patterns, and variability in hospitalization duration, conceptualized as a complex and clinically determined outcome.

Despite the established relevance of emotion regulation and attachment in suicidality and treatment outcomes, the specific impact of these variables on hospitalization duration among suicidal adolescents has not been previously explored. This study addresses this gap. We hypothesized that greater emotion regulation difficulties would predict longer hospitalization duration, and that this effect would remain observable even after accounting for additional contextual variables, such as family environment, discharge destination (home versus out-of-home placement), and whether patients were receiving psychotropic medication. Additionally, we hypothesized that both anxious and avoidant attachment styles would predict longer hospitalizations and potentially would mediate the effects of emotion regulation on hospitalization duration. We further posited that these associations would persist independently of psychiatric symptoms of depression and anxiety, and the severity of suicidal ideation at admission.

## 2. Materials and Methods

### 2.1. Participants

#### Sample and Procedure

To determine the required sample size, we used G*Power (version 3.1.9.7) with the following parameters: a type I error rate of 5%, a statistical power of 80%, and an assumed bivariate correlation of *r* = 0.3. This calculation indicated that a sample size of 67 participants was required.

Following ethical approval from the Institutional Ethics Committee and the National Ministry of Health, adolescents were recruited as part of an ongoing longitudinal study conducted in a tertiary medical center, examining the efficacy of a novel clinical intervention to reduce suicidality in youth: the As Safe As Possible (ASAP) intervention for youth at risk of suicide [50]. The study was conducted in accordance with the Declaration of Helsinki. Written informed consent was obtained from all participants; for those under the age of 18, written consent was also obtained from their legal guardians. While the analysis of the ASAP intervention is beyond the scope of this manuscript, the present report focuses on a subset of data collected during psychiatric hospitalization as part of a larger ongoing research program.

Inclusion criteria included adolescents aged 12–18 years admitted to an inpatient child and adolescent psychiatric setting due to a suicide attempt within the month prior to study recruitment or current suicidal intent. Exclusion criteria were the presence of sensorimotor or intellectual disability, bipolar disorder, psychosis, previous diagnosis of head trauma, or organic sleep disorder.

Recruitment occurred during the admission process to the inpatient unit. During the initial intake meeting, one of the principal investigators approached all adolescents who met the inclusion criteria and invited them to participate in the study. At the same meeting, parents received a detailed explanation of the study procedure and provided written informed consent. Approximately 80% of eligible patients and their parents agreed to participate.

The psychiatric inpatient unit in which the study participants were treated follows a structured therapeutic daily schedule based on standardized clinical routines delivered by licensed professionals, including clinical social workers, psychologists, and child and adolescent psychiatrists. Each patient routinely received a twice-weekly individual psychotherapy, alongside a parallel parental-guidance process and a twice-weekly psychiatric follow-up. In addition, patients participated in various therapeutic group interventions, including dynamic psychotherapy, art therapy, and Dialectical Behavior Therapy (DBT). The daily schedule included regular meals, fixed wake-up times, and attendance at an on-site specialized school staffed by special education teachers and located within the ward. Each patient’s case was reviewed in multidisciplinary twice-weekly team meetings, where treatment progress, inpatient interventions, and an individualized post-discharge treatment plan were discussed and revised as needed.

The sample consisted of 79 adolescents aged 12–18, predominantly female (87.3%), with an average age of 15.35 years (SD = 2.03). Most participants (74.68%) reported that their parents were married. After signing informed consent, baseline assessments were conducted during admission within the first week of hospitalization. Psychiatric diagnoses were established using the validated K-SADS [51], administered to both adolescents and their parents, and confirmed by the inpatient multidisciplinary team, including a senior psychiatrist and a clinical psychologist. Discharge decisions were determined through structured multidisciplinary team meetings held twice weekly, including a psychiatrist, a psychologist, and a social worker, and were based on comprehensive clinical evaluation, in addition to C-SSRS scores, with a score of 3 or lower on suicidal ideation severity indicating readiness for discharge. Discharge planning was conducted in advance, and decisions were supported by ongoing team review to ensure clinical stability and continuity of care. Additional conditions for discharge included the availability of an appropriate follow-up treatment setting that could provide continuity of care immediately after discharge.

The average length of hospitalization was approximately three months. Sample sociodemographic characteristics, including suicidality scores and diagnostic information, are presented in Table 1.

### 2.2. Measures

The Difficulties in Emotional Regulation Scale (DERS) [52] is a 36-item self-assessment tool designed to measure clinically relevant emotion regulation difficulties by assessing challenges in the following areas: (a) awareness and understanding of emotions, (b) acceptance of emotions, (c) engagement in goal-directed behavior and impulse control in the presence of negative emotions, and (d) the ability to regulate emotions using effective strategies. Participants rated the frequency with which each statement applied to them on a scale from 1 (almost never) to 5 (almost always). Higher total scores indicate greater emotion regulation difficulties, whereas lower scores reflect better emotion regulation abilities. In the present sample, DERS demonstrated good internal reliability (Cronbach’s α = 0.86).

The Experience in Close Relationships Scale (ECR) [17] is a 36-item self-report measure assessing two dimensions of attachment: anxiety (fear of rejection) and avoidance (discomfort with closeness). Items are rated on a 7-point Likert scale, and subscale scores are calculated as the mean of relevant items. The ECR has demonstrated high internal consistency and strong construct validity. This study used a translated version of the ECR, which has demonstrated validity and reliability in previous research [53,54]. Although originally developed for adults, versions of the ECR and related scales have been psychometrically validated in adolescent samples, demonstrating adequate reliability and construct validity in this age group [55,56,57]. Participants were instructed to complete the questionnaire with reference to their parents as primary attachment figures, rather than romantic partners. In the current study, the ECR demonstrated excellent internal consistency for the Avoidance subscale (Cronbach’s α = 0.91) and good internal consistency for the Anxiety subscale (Cronbach’s α = 0.86).

The Screen for Child Anxiety-Related Emotional Disorders (SCARED) [58] is a self-report questionnaire designed to assess anxiety symptoms in children and adolescents aged 8 to 18 years. The scale consists of 41 items, each rated on a 3-point scale: 0 (almost never), 1 (sometimes), and 2 (often). SCARED measures five key categories of anxiety disorders: panic/somatic symptoms, generalized anxiety, separation anxiety, social phobia, and school phobia. In the current study, the SCARED demonstrated excellent internal consistency (Cronbach’s α = 0.96).

The Patient Health Questionnaire (PHQ9) [59] is a self-report, nine-item questionnaire designed to screen for depression. Each item corresponds to one of the nine diagnostic criteria for major depressive disorder as outlined in the DSM-V. Responses are rated on a 4-point scale: 0 (not at all) to 3 (nearly every day). A total score is calculated, with higher scores indicating greater depressive symptom severity. A diagnosis of major depression is suggested if five or more items are rated as occurring on at least “more than half the days” over the past two weeks, provided that one of the symptoms is either depressed mood or anhedonia. The PHQ-9 exhibited excellent internal consistency in the present sample (Cronbach’s α = 0.94).

The Columbia–Suicide Severity Rating Scale (C-SSRS) [60] is a clinician-administered semi-structured interview, designed to assess the full range of suicidal ideation and behavior. It evaluates the severity and intensity of suicidal thoughts, as well as actual, aborted, and interrupted suicide attempts.

Schedule for Affective Disorders and Schizophrenia for School-Age Children Present and Lifetime Version (K-SADS) [51] is a semi-structured interview, based on the DSM-5. It is one of the most commonly used tools for assessing the mental health of children and adolescents, and it has been widely used in clinical and research settings. Structured psychiatric assessments were conducted through semi-structured interviews with participants and their parents by using the DSM-5 Hebrew version of the affective, PTSD, and anxiety modules of the K-SADS.

The Parental Bonding Instrument (PBI) [61] is a 25-item self-report questionnaire designed to assess perceived parenting behaviors during the first 16 years of life. The PBI evaluates two key dimensions for each parent: care (warmth and affection versus coldness and neglect) and overprotection (control and intrusion versus autonomy granting). Items are rated on a 4-point Likert scale, and subscale scores are calculated separately for mothers and fathers.

Hospitalization duration was measured using medical records. The length of stay was calculated as the number of days between the entry and discharge dates.

A demographic questionnaire was administered to collect relevant background information from participants. This included variables such as age, gender, parental marital status, and other sociodemographic characteristics.

### 2.3. Statistical Analysis

Descriptive statistics were generated to summarize the dataset. Categorical variables were reported using frequencies and percentages (*n*/%), allowing for a clear representation of the distribution of each category. For continuous variables, means and standard deviations (M ± SD) were calculated to provide insights into central tendency and variability. We computed Pearson correlations between study variables.

To address missing data within the model, we applied the Full Information Maximum Likelihood (FIML) method [62], a robust approach that allows for parameter estimation using all available data. Unlike traditional missing data techniques such as listwise or pairwise deletion, FIML incorporates all observed data points and estimates missing values within the likelihood function, reducing bias and improving the efficiency of parameter estimates. By employing FIML within the SEM framework, we ensured that the analysis was conducted with minimal data loss, maintaining the integrity and statistical power of the findings.

To examine the potential mediation effects of emotional regulation (independent variable) on hospitalization duration (dependent variable), we employed Path analysis within the framework of Structural Equation Modeling (SEM) [63]. This analytical approach allows for the assessment of both direct and indirect effects between variables, while also accounting for measurement errors and inter-variable relationships. To ensure the robustness of the model, we adjusted for key demographic covariates, both gender and age, which could potentially confound the relationship between emotion regulation and hospitalization duration. In addition, we controlled for the suicidal behavior score at baseline.

The same approach was applied to the mediation model exploring the relationship between emotional regulation, depression, anxiety and hospitalization duration, allowing us to assess how depression and anxiety might mediate the link between emotional regulation and hospitalization duration.

The following indices were used to evaluate the model: chi-squared, which is acceptable when the value is not significant; the goodness-of-fit index (GFI); the comparative fit index (CFI) (adequate values—above 0.90, excellent fit—above 0.95); and the root mean square error of approximation (RMSEA) (adequate values—less than 0.08, excellent fit—less than 0.06). SEM was conducted using IBM SPSS Amos, Version 28 [63].

Indirect effects were evaluated using bootstrapping with 5000 resamples. Significance of the indirect effects was determined using 95% percentile bootstrap confidence intervals, whereby an effect was considered significant if the interval did not contain zero [64].

## 3. Results

As shown in Table 2, hospitalization duration was positively associated with emotion regulation difficulties (DERS total; *r* = 0.326, *p* < 0.001), suggesting that greater emotion regulation difficulties predicted longer hospitalization stays. In contrast, hospitalization duration was negatively associated with avoidant attachment (*r* = −0.171, *p* < 0.05), indicating that greater avoidant attachment predicted shorter hospitalization stays.

### 3.1. Multivariate Regression Model Predicting Hospitalization Duration

The results of the multiple regression analysis predicting length of hospitalization are presented in Table 3.

The model identified two significant predictors: age at enrollment and baseline emotion regulation difficulties (DERS total). Age at the time of enrollment was negatively associated with hospitalization duration, such that older adolescents had significantly shorter stays (β = −0.30, *p* < 0.001). In contrast, baseline emotion regulation difficulties (DERS total) were positively associated with hospitalization, indicating that greater emotion regulation difficulties predicted longer inpatient duration (β = 0.49, *p* < 0.001). No other predictors have reached significance.

Baseline DERS emerged as the strongest predictor, such that each 1-point increase in DERS was associated with an additional 2.06 days of hospitalization, indicating that youths with greater emotion regulation difficulties required substantially longer inpatient care. Age at enrollment showed the opposite pattern: each additional year of age predicted a 12.45-day reduction in length of stay, meaning younger adolescents remained hospitalized significantly longer even after controlling for symptom severity. Other predictors showed smaller or non-significant contributions. Sex was not associated with meaningful changes in LOS, with males averaging about 14 fewer days than females, though this effect was not statistically reliable. Attachment avoidance (−17.72 days per 1-unit increase), attachment anxiety (−16.28 days), baseline PHQ-9 depressive symptoms (−0.11 days), and baseline SCARED anxiety symptoms (+0.33 days) each showed very small or inconsistent effects, none of which reached statistical significance. Overall, the regression indicates that hospitalization duration is most strongly shaped by patients’ emotion regulation capacity and age, whereas baseline anxiety, depressive symptoms, or attachment patterns do not appear to contribute meaningfully to predicting LOS in this sample.

This model controlled for PBI scores and medications (“yes” vs. “no” psychotropic medication). No significant associations were found with hospitalization duration.

To account for potential structural influences on hospitalization duration, we conducted an additional analysis including discharge destination (return home vs. therapeutic out-of-home placement) as a control variable. The inclusion of this variable did not alter the pattern or significance of the primary findings.

### 3.2. Mediation Model 1. Emotion Regulation, Attachment, and Hospitalization Duration

**Figure 1:**
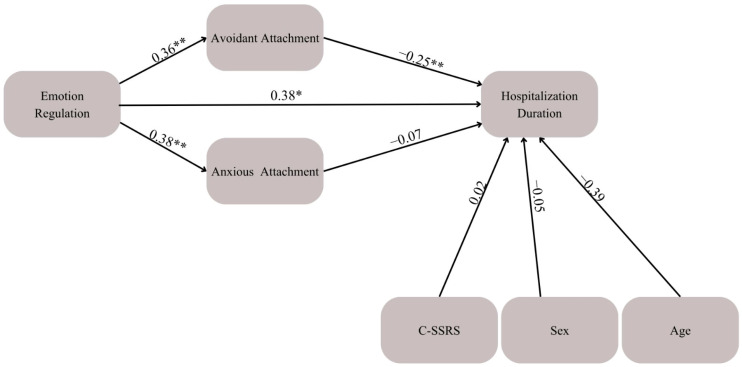
Mediation Model examining the associations between emotion regulation, avoidant and anxious attachment, and hospitalization duration. Standardized path coefficients are presented. Age, sex, and baseline suicidality (C-SSRS) were included as covariates. * *p* < 0.05, ** *p* < 0.01.

This model demonstrated acceptable goodness-of-fit indices: χ^2^ (11) = 11.47, *p* = 0.163; CFI = 0.93; GFI = 0.96; RMSEA = 0.08; SRMR = 0.05. A significant direct effect was found between emotion regulation and hospitalization duration (β = 0.38, *p* < 0.05), indicating that greater emotion regulation difficulties predict longer hospitalization. Greater emotion regulation difficulties was also significantly associated with both avoidant attachment (β = 0.36, *p* < 0.01) and anxious attachment (β = 0.38, *p* < 0.01). In turn, higher avoidant attachment was associated with shorter hospitalization duration (β = −0.25, *p* < 0.01), whereas no significant effect was found for anxious attachment (β = −0.07, *p* = 0.81). A significant mediation effect was found between emotion regulation and hospitalization duration via avoidant attachment (β = −0.117, *p* = 0.04, 95% CI [−0.21, −0.13]), suggesting that for individuals whose greater emotion regulation difficulties leads to avoidant attachment, hospitalization is shorter, as shown in Figure 1. To note, this model controlled for medication and PBI scores.

Examining the opposite model yielded relatively poor results: χ^2^ (7) = 20.32, *p* = 0.06; CFI = 0.83; GFI = 0.84; RMSEA = 0.09; SRMR = 0.07.

Following the results presented in Figure 1, we sought to rule out an alternative explanation for these effects. Given the well-established associations between emotion regulation difficulties and psychopathologies such as depression, anxiety, and suicidality, we conducted an additional model to examine whether these symptoms could account for the observed effects. Specifically, we tested whether depression and anxiety symptoms, as well as the level of suicidal ideation and behavior at admission, independently predicted hospitalization duration or mediated the relationship between emotion regulation and length of stay. This allowed us to determine whether the predictive value of emotion regulation reflected a distinct mechanism or was merely a byproduct of underlying psychopathology.

### 3.3. Mediation Model 2. Emotion Regulation, Depression/Anxiety, and Hospitalization Duration

**Figure 2:**
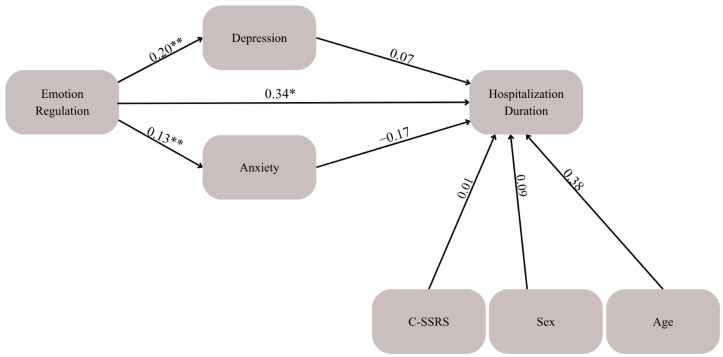
Mediation Model examining the associations between emotion regulation, baseline depression and anxiety, and hospitalization duration. Standardized path coefficients are presented. Age, sex, and baseline suicidality (C-SSRS) were included as covariates. * *p* < 0.05, ** *p* < 0.01.

Additional analyses were conducted to test the possible effect of psychopathology on hospitalization duration. DERS scores were positively correlated with depression at admission (β = 0.20, *p* < 0.01) and anxiety at admission (β = 0.13, *p* < 0.05). However, no significant association was found between anxiety and hospitalization duration (β = −0.17, *p* = 0.10), nor between depression and hospitalization duration (β = 0.07, *p* = 0.66). Additionally, a mediation model testing the indirect effect of emotion regulation on hospitalization via anxiety yielded a non-significant result (β = −0.09, *p* = 0.07, 95% CI [−0.11, 0.10]), indicating that anxiety did not mediate the relationship between emotion regulation difficulties and hospitalization duration as shown in Figure 2.

To further examine the confounding effects, we conducted an additional analysis including suicidality severity at admission (C-SSRS total ideation and behavior scores and intensity score) as a covariate in the mediation model. This analysis examined whether the observed association between emotion regulation and hospitalization duration remained after accounting for baseline suicidality. The pattern of results remained consistent: greater emotion regulation difficulties significantly was associated with longer hospitalization duration, and the mediation effect via avoidant attachment remained significant. Suicidality did not significantly predict hospitalization duration (β = −0.05, *p* = 0.48) and did not alter the strength or direction of the primary effects.

We examined whether the type of discharge setting was associated with the primary study outcomes. No significant associations were observed between discharge setting and hospitalizations (*p* = 0.509), DERS scores (*p* = 0.590), SCARED scores (*p* = 0.765), or PHQ-9 scores (*p* = 0.656).

## 4. Discussion

This study aimed to examine the impact of emotion regulation and attachment styles on the length of psychiatric hospitalization among adolescents admitted following a suicide attempt or due to current suicidal ideation. The results supported our first hypothesis, indicating that greater emotion regulation difficulties predicted longer hospitalization duration. However, our second hypothesis regarding attachment style was not supported, as avoidant attachment was associated with shorter hospitalization duration, contrary to expectations. These findings should be considered in light of prior evidence suggesting that prolonged hospitalizations may increase adolescents’ exposure to maladaptive peer dynamics, such as symptom contagion and behavioral modeling, potentially amplifying distress and undermining treatment efficacy [40,42].

It is important to acknowledge that hospitalization duration is not determined solely by psychological variables. Institutional policies, bed availability, and healthcare system structures may influence discharge timing. However, in the present setting, discharge decisions were based on clinical stabilization criteria, and there was no systematic pressure related to insurance or bed turnover. Moreover, discharge destination was included in the analyses to account for cases awaiting out-of-home placement, and this did not alter the pattern of findings.

Consistent with our first hypothesis, greater emotion regulation difficulties predicted longer hospitalization duration. This result aligns with the existing literature emphasizing the central role of emotion regulation difficulties in adolescent psychopathology and treatment processes [46]. However, contrary to our second hypothesis, attachment style predicted hospitalization duration in an unexpected direction. Although anxious attachment was associated with greater emotion regulation difficulties, it did not predict hospitalization duration, which is in contrast with prior research linking anxious attachment to heightened psychological distress and increased support-seeking behaviors [20]. In contrast, avoidant attachment demonstrated an inverse relationship; despite its association with greater emotion regulation difficulties, avoidance was associated with shorter hospitalization duration. One possible interpretation of this finding is that adolescents with higher levels of avoidant attachment may be more inclined to suppress emotional distress and limit engagement within therapeutic relationships. In this context, shorter hospitalization could reflect differences in treatment engagement. However, this explanation remains speculative, as the present study did not include direct measures of therapeutic alliance or treatment participation. Alternative explanations should also be considered. For example, avoidant attachment may be associated with more restricted emotional expression or reduced help-seeking, which could influence how stabilization and readiness for discharge are perceived within the inpatient context. In this case, shorter hospitalization may reflect differences in interpersonal presentation rather than early therapeutic disengagement.

To address the possibility that the relationship between emotion regulation and hospitalization duration was simply a reflection of underlying psychopathology, we conducted additional analyses. Although emotion regulation difficulties were associated with higher levels of depression, anxiety, and suicidality at admission, none of these clinical symptoms significantly predicted hospitalization duration. Moreover, neither anxiety, nor depression, nor suicidal ideation and behavior severity mediated the relationship between emotion regulation and hospitalization length. These results suggest that while greater emotion regulation difficulties are indeed linked to greater psychopathology, it is not these symptoms per se that account for differences in hospitalization duration. Instead, emotion regulation appears to have an independent and direct contribution to treatment length.

Further insight was provided by the mediation model, which indicated that the influence of emotion regulation on hospitalization duration was partially explained by avoidant attachment. Specifically, greater emotion regulation difficulties were associated with higher levels of avoidant attachment, which, in turn, predicted shorter hospitalizations. These findings underscore the importance of considering both emotion regulation capacities and attachment patterns when evaluating treatment engagement and predicting hospitalization outcomes. Traditional symptom-based measures may not fully capture the interpersonal and emotional processes that shape inpatient care trajectories in adolescents. However, given the sample size and model complexity, these mediation findings should be considered preliminary and interpreted with caution. Importantly, these associations do not imply direct causal effects but rather reflect potential interactions between emotional, interpersonal, and clinical decision-making processes.

The proposed mediation model should be interpreted with caution. Both emotion regulation difficulties and attachment orientations were assessed at baseline during hospitalization; therefore, the model does not imply developmental or causal precedence. Although attachment patterns are typically conceptualized as developmentally antecedent to emotion regulation capacities, the present analysis examined their functional interplay within the inpatient context. In this framework, attachment orientations were not assumed to be shaped by emotion regulation, but rather to influence how regulatory difficulties relate to hospitalization duration.

The present findings have important clinical implications. Difficulties in emotion regulation may contribute to longer hospitalization by limiting adolescents’ ability to tolerate distress and engage effectively in treatment during acute suicidal crises. Experimental evidence demonstrates that different regulation strategies distinctly affect emotional and physiological responses, and that attachment orientations moderate these effects [65]. These findings are consistent with clinical approaches that directly target regulatory capacities. Dialectical Behavior Therapy (DBT), for example, focuses on strengthening emotion regulation and distress tolerance skills and has demonstrated efficacy in reducing suicidality among adolescents [66]. In addition, attachment-informed interventions, such as Attachment-Based Family Therapy (ABFT) and Mentalization-Based Therapy (MBT), aim to enhance attachment security and reflective functioning, thereby supporting regulatory capacities [67,68]. Together, these approaches align with the current results, underscoring the importance of integrating skills-based and attachment-focused interventions within inpatient treatment for suicidal youth.

### Limitations

While this study provides valuable contributions to understanding factors influencing hospitalization duration among suicidal adolescents, several limitations should be noted. First, although the study utilized a longitudinal design, emotion regulation and attachment were assessed only at the beginning of hospitalization. Future research would benefit from tracking these variables across multiple time points, including post-discharge, to capture their potential fluctuations and dynamic impact on treatment outcomes. Second, although the sample size met the requirements of the power analysis, it was relatively small and predominantly female. Further studies with larger and more gender-balanced samples may help validate these findings and explore potential gender differences in emotion regulation, attachment, and treatment trajectories. Although the ECR has been used in adolescent samples, it was originally developed for adults, and future research may benefit from incorporating attachment measures specifically designed for adolescents. In addition, several clinical variables that may influence discharge decisions—such as medication response, level of participation in psychotherapy, and detailed aspects of family functioning—were not systematically measured in the present study and therefore could not be included in the analytic model. Importantly, discharge destination (return home vs. therapeutic out-of-home placement), which partially reflects post-discharge planning and aspects of family context, was included as a covariate and did not alter the pattern of findings. Nevertheless, the absence of additional clinical process variables may limit the comprehensiveness of the model and introduce the possibility of omitted variable bias. Future research should incorporate more detailed treatment and contextual measures to better account for variability in hospitalization duration.

Although family context and discharge destination were considered in the analyses, other clinical process variables were more difficult to account for. In particular, no formal measure was used to quantify treatment engagement. Nevertheless, all patients participated in multidisciplinary care, including individual and group psychotherapy as well as regular psychiatric follow-up, and none exhibited clear non-cooperation with treatment. In addition, whether patients were receiving psychotropic medication was also included in the analyses. However, medication response could not be meaningfully assessed, as it is influenced by multiple factors and cannot be reliably isolated in the present sample.

Although additional clinical process variables—such as medication response, therapeutic engagement, and more detailed indices of family context—were not included in the present model, emotion regulation difficulties remain a theoretically and clinically central construct in adolescent mental health and suicidality. Examining emotion regulation within a clinically acute inpatient population of adolescents hospitalized following suicidal crises therefore provides meaningful insight into variability in hospitalization duration. Future research should aim to replicate and extend these findings by incorporating additional treatment-process and contextual variables to further elaborate the model.

Moreover, the finding that avoidant attachment predicted shorter hospitalization was contrary to our initial hypothesis. The explanatory accounts proposed are therefore exploratory, and replication in independent samples is needed to clarify this association.

The mediation analyses were exploratory in nature and were not included in the original power calculation, which was designed for the primary regression-based hypotheses. Detecting indirect effects with 0.80 power for small-to-medium path coefficients typically requires sample sizes exceeding the current study’s *N* of 79 [69]. The mediation findings should therefore be considered preliminary and interpreted with caution, particularly for small effects, pending replication in larger samples. Larger samples will be required to precisely estimate indirect effects and to support confirmatory mediation testing.

Finally, our interpretation assumed that longer hospitalization represents an undesired outcome due to potential side effects and the disruption of normative developmental processes. However, the clinical meaning of hospitalization duration in adolescent populations remains understudied and warrants further empirical investigation.

While the average length of hospitalization in our sample was approximately three months, which is longer than in many other countries and inpatient care units worldwide, the associations observed in this study were statistically significant, suggesting that these findings are clinically relevant and important to share with the international community. Hospitalizations were for acute crises, and discharge was contingent on the availability of an appropriate follow-up care setting, such as outpatient services, family support, or residential treatment. This requirement may partly explain the longer length of stay compared with other countries and inpatient programs. Future studies could examine similar-length inpatient programs in other countries to further contextualize these findings.

## 5. Conclusions

This study is the first, to our knowledge, to examine the combined effects of emotion regulation difficulties and attachment styles on hospitalization duration among suicidal adolescents. The findings indicate that emotion regulation difficulties serve as a robust and independent predictor of longer inpatient stays, beyond the influence of depression, anxiety, and suicidality. Additionally, avoidant attachment was associated with shorter hospitalization, suggesting that interpersonal dynamics significantly shape the course of inpatient treatment, potentially through reduced therapeutic engagement.

From a research perspective, these findings underscore the central role of emotion regulation in the psychopathology of suicidal adolescents. Beyond its clinical relevance, emotion regulation difficulties appear to be a key transdiagnostic factor influencing treatment trajectories. Future research should further explore the mechanisms through which emotion regulation difficulties affect inpatient processes and outcomes, potentially incorporating longitudinal designs and multi-informant assessments. Examining interactions with other developmental factors, such as peer relationships, family dynamics, and neurobiological markers, may also enrich our understanding of emotion regulation as a target for early intervention.

These novel findings emphasize the importance of systematically incorporating assessments of emotional and relational functioning into clinical evaluations and treatment planning for adolescents in psychiatric crisis. Adolescents with substantial greater emotion regulation difficulties may particularly benefit from interventions that enhance emotional awareness, acceptance, and adaptive regulatory strategies, such as cognitive reappraisal. For those with avoidant attachment patterns, clinicians should carefully monitor therapeutic engagement and discharge readiness, as early discharge may reflect emotional disengagement rather than meaningful clinical progress.

By introducing a new perspective on predictors of hospitalization outcomes in suicidal youth, this study underscores the need for personalized, emotion-focused, and relationally informed approaches in inpatient psychiatric care. Such strategies may optimize treatment effectiveness, support recovery, and contribute to more clinically informed decision-making regarding hospitalization duration.

## Figures and Tables

**Table 1:** Sample characteristics and descriptive statistics (*N* = 79).

Variable	*N*	%	M	SD
**Sex**				
Female	69	87.34		
Male	10	12.66		
Age			15.35	2.03
**Country of origin**				
Israel	72	91.14		
Other countries	7	8.86		
**Level of religiosity**				
Secular	38	48.1		
Traditional	7	8.86		
Religious	20	25.32		
Ultra-orthodox	8	10.13		
**Hospitalization duration (days)**			126.29	87.01
**Active psychiatric diagnosis ^a^**				
Major Depressive Disorder (MDD)	38	48.1		
Post-Traumatic Stress Disorder (PTSD)	4	5.06		
Attention-Deficit/Hyperactivity Disorder (ADHD)	4	5.06		
Social Anxiety	1	1.27		
Anorexia	7	8.86		
Generalized Anxiety Disorder (GAD)	4	5.06		
Obsessive–Compulsive Disorder (OCD)	4	5.06		
Borderline Personality Disorder (BPD)	2	2.53		
Trichotillomania	2	2.53		
Tic Disorder	1	1.27		
Other	6	7.59		
**Columbia- Suicide Severity Rating Scale (C-SSRS)**				
Intensity Score			14.94	4.13
Behavior Score			3.26	2.12

^a^ Missing values were observed for some variables (Level of religiosity: *n* = 6, 7.55%; Active psychiatric diagnosis: *n* = 6, 7.59%).

**Table 2:** Correlation matrix.

	**1**	**2**	**3**	**4**	**5**	**6**
**Avoidant Attachment**	-	-	-	-	-	-
**Anxious Attachment**	0.356 **	-	-	-	-	-
**PHQ9 score (depression)**	0.290 **	0.425 **	-	-	-	-
**SCARED (Anxiety)**	0.569 **	0.524 **	0.673 **	-	-	-
**DERS (Emotion Regulation)**	0.358 **	0.381 **	0.255 **	0.426 **	-	-
**Hospitalization Duration**	−0.171 *	−0.023	−0.042	−0.071	0.326 **	-

Note: *p* < 0.05 * *p* < 0.01 **.

**Table 3:** Regression coefficients predicting hospitalization duration.

Predictor	B	SE B	Β	t	*p*
**Constant**	274.71	181.08	-	1.52	0.14
**Sex (Male)**	22.95	33.78	0.08	0.68	0.5
**Age at enrollment**	−12.45	6.11	−0.3	−2.04	0.05
**Avoidance (ECR)**	−17.72	10.54	−0.25	−1.68	0.1
**Anxiety (ECR)**	−16.28	13.61	−0.18	−1.2	0.24
**Baseline PHQ-9**	−0.11	1.99	−0.01	−0.05	0.96
**Baseline SCARED**	0.33	1.05	0.06	0.31	0.76
**Baseline DERS**	2.06	0.63	0.49	3.27	0
**Medication (yes)**	−35.17	27.48	−0.17	−1.28	0.21
**PBI Father Care**	0.13	3.67	0.01	0.04	0.97
**PBI Father Over-Protective**	−0.56	2.07	−0.05	−0.27	0.79
**PBI Mother Care**	−1.88	3.95	−0.07	−0.47	0.64
**PBI Mother Over-Protective**	−0.33	1.81	−0.03	−0.18	0.86

ECR—The Experience in Close Relationships Scale, PHQ-9—The Patient Health Questionnaire, SCARED—The Screen for Child Anxiety-Related Emotional Disorders, DERS—The Difficulties in Emotional Regulation scale, PBI—Parental Bonding instrument.

## Data Availability

The data presented in this study are available on request from the corresponding author. The data are not publicly available due to privacy restrictions.
